# Nutrient Content, Organoleptic Quality, and Shelf Life of *Sagon* Substitute From *Lindur* (*Bruguiera gymnorrhiza* L.) and Soybean Flour (*Glycine max* L.), as an Alternative Emergency Food

**DOI:** 10.3389/fnut.2022.878539

**Published:** 2022-04-19

**Authors:** Diana Nur Afifah, Yesi Pratama Aprilia Ningrum, Tazkiah Syahidah, Nuryanto Nuryanto, Fitriyono Ayustaningwarno, Denny Nugroho Sugianto

**Affiliations:** ^1^Department of Nutrition Science, Faculty of Medicine, Diponegoro University, Semarang, Indonesia; ^2^Center of Nutrition Research (CENURE), Faculty of Medicine, Diponegoro University, Semarang, Indonesia; ^3^Department of Oceanography, Faculty of Fisheries and Marine Science, Diponegoro University, Semarang, Indonesia

**Keywords:** nutrition content, organoleptic quality, shelf life, *sagon*, *lindur*, emergency food

## Abstract

Lindur (*Bruguiera gymnorrhiza* L.) fruit as a mangrove species has not been widely developed. However, the combination of *lindur* fruit functional food with soybean flour has high carbohydrates and protein potential to serve as an additional food product in emergency conditions. Therefore, this study aims to evaluate the nutritional content, dietary fiber, organoleptic quality, and *sagon* shelf-life estimation of *lindur* and soybean flour, in accordance with emergency food quality requirements. The shelf life was determined using the Accelerated Shelf-life Testing (ASLT) method with the Arrhenius equation, where the sample was stored in an incubator at a temperature of 27, 37, as well as 47°C for 30 days, and the water content, peroxide number, and free fatty acid content were tested every 5 days. In addition, formulation P1 comprising 45% *lindur* flour and 35% soybean flour was discovered to be the best formulation, with a composition of 251.5 kcal/bar of energy, 6.3 g of fat, 4.4 g of protein, 30 g of carbohydrates, 15.79% dietary fiber, 1.84% ash content, and 4.03% water content. Therefore, the nutritional content of *sagon* substitution for *lindur* flour is in accordance with the emergency food quality requirements, except for the protein content. Also, the P2 *sagon* sample’s shelf life was estimated to be 37 days in polypropylene plastic packaging at a room temperature of 27°C.

## Introduction

Indonesia’s territory lies between the three tectonic plates and has many volcanoes, making the country vulnerable to natural disasters (1). According to the Indonesian National Disaster Management Agency, 1944 natural disasters were recorded in Indonesia between January and September 2020. These occurrences led 3.8 million victims to take refuge in emergency housing facilities, where emergency foods with the capacity to fulfill human energy needs, are urgently required (2).

The term “emergency food” is a special type of food suitable for consumption during or after emergency conditions to fulfill the human energy need of 2,100 kcal/day, and ought to comprise 35–45% fat, 10–15% protein, and 40–50% carbohydrates (3). A good example of an emergency food product is *sagon*, traditional Indonesian food with a sweet taste, dry texture, low water content, and consequently, long shelf life (4). Furthermore, *sagon* is suitable for consumption for all ages and is, therefore, highly suitable for use as snacks or emergency food.

*Lindur* (Bruguiera gymnorrhiza) is a mangrove fruit rich in carbohydrates and is often used by coastal communities as an alternative local food choice during the lean season, to prevent food insecurity (5). In addition, the nutritional composition of *lindur* is 32.91% carbohydrates, 0.79% fat, 2.11% protein, 1.29% ash content, and 62.92% water (6).

The protein value of emergency food products is often increased through the addition of soybeans (*Glycine max* L.). Soybean flour has a high protein content of 34.8% and a fiber content of 3.2% per 100 g (7). Furthermore, *sagon* contains coconut which has a high-fat content of up to 33.49%, and this tends to affect the product shelf life as high-fat content increases the risk of product rancidity (8).

The purpose of this research was to determine the nutritional content (protein, fat, carbohydrate, water content, ash content, dietary fiber, and energy), organoleptic quality, and shelf life of *sagon* made from *lindur* flour and soybean flour as an alternative emergency food capable of meeting the nutritional needs of natural disaster victims. The key parameters of color, aroma, taste, and texture are used to define organoleptic quality. Water content, peroxide number, and fatty acid content are all measured to determine shelf life.

## Materials and Methods

### *Sagon* Formulation

#### The Raw Material

Ripe *lindur* fruit (*B. gymnorrhiza* L.) from Mangunharjo Village, Mangkang Kulon Village, Tugu District, Semarang has a green-brown color with brown spots (15–25 cm). Soybean flour, glutinous rice flour, grated coconut, sugar, and salt are among the other ingredients.

#### *Sagon* Procedure

Roast the glutinous rice flour, *lindur* flour, soybean flour, and grated coconut for 10 min at 70°C. In a mixing bowl, combine the sugar, salt, grated coconut, *lindur* flour, soybean flour, and roasted glutinous rice flour. Stir until evenly combined. The dough is then formed into a *sagon* shape using a *sagon* mold. *Sagon* in the oven for 20 min at 150°C. The ripe *sagon* is removed from the mold and allowed to cool to room temperature (25°C) before being vacuum sealed in plastic. After vacuuming, the *sagon* is stored in a storage container for 2 days before being analyzed the following day.

This study formulated *sagon* using *lindur* and soybean flour. A total of four formulations (*lindur*: soybean flour): P1 (45: 35%), P2 (40: 40%), P3 (35: 45%), P4 (30: 50%), were created in triplicates. Table 1 shows the composition of ingredients used in each formulation.

**TABLE 1:** Formulation of *sagon* from *lindur* and soybean flour.

*Sagon* ingredients	Quantity of ingredients (100 g)			
	P1	P2	P3	P4
*Lindur* flour	15	13.33	11.67	10
Soybean flour	11.67	13.33	15	16.67
Glutinous rice flour	6.67	6.67	6.67	6.67
Grated coconut	38.09	38.09	38.09	38.09
Sugar	19.05	19.05	19.05	19.05
Oil	9.52	9.52	9.52	9.52
Salt	0.57	0.57	0.57	0.57

*P1 (lindur 45%: soybean flour 35%), P2 (lindur 40%: soybean flour 40%), P3 (lindur 35%: soybean flour 45%), P4 (lindur 30%: soybean flour 50%).*

### Nutritional Content

The *sagon* samples’ analysis of water content was determined by the dry cup method [AOAC (9)], Ash content analysis refers to ash content analysis. Determination of fat content is carried out by the Soxhlet method [AOAC (9)].

The protein content was determined using the micro-Kjeldahl method [AOAC (9)]. The general concept behind this analysis is to determine protein by oxidizing carbonaceous materials and converting nitrogen into ammonia. After that, the ammonia reacts with the excess acid to form arnonium sulfate. The ammonia is evaporated after the solution becomes alkaline in order to be absorbed in the boric acid solution. HCL titration was used to determine the amount of nitrogen present. The Kjeldahl method, which includes destruction, distillation, and titration, is used to determine protein content. After obtaining the titration volume, use the formula to compute the protein content:


$$\text{Nitrogen}\text{C}\mathrm{ontent}\left(\%\right)=\frac{\begin{array}{c} {}[TitrationVolume\times HClNormality\left(0.02N\right)\\ \times NitrogenAtomicWeight\left(14.008\right)] \end{array}}{S\,a\,m\,p\,l\,e\,W\,e\,i\,g\,h\,t\,\left(M\,i\,l\,l\,i\,g\,r\,a\,m\,s\right)}\times 100\%$$

Proteincontent(%wb)=Nitrogencontent×conversionfactor(6,10)

The carbohydrate content was analyzed by difference, which was the result of reducing from 100% with water content, ash content, protein content, and fat content, so that the carbohydrate content was dependent on the reduction factor. This is due to the fact that carbohydrates have a large influence on other nutrients [AOAC (9)]. This is because carbohydrates are very influential on other nutrients. The energy content is determined using the total calorie calculation method, as shown in Equation 1. Protein has a 4 kcal/g energy value, fat has a 9 kcal/g energy value, and carbohydrates have a 4 kcal/g energy value.


(1) Total⁢calories=(4×PC)+(4×CC)+(9×FC)

where PC, protein content (g); CC, carbohydrate content (g); FC, fat content (g).

Subsequently, the Multienzyme method was used to determine total dietary fiber [AOAC (9)].

### Organoleptic Test

This test was carried out by a moderately trained panel of 30 undergraduate students from Nutrition Science of Diponegoro University’s. Each panelist was given four samples of *sagon*. The samples given were P1 (45: 35%), P2 (40: 40%), P3 (35: 45%), and P4 (30: 50%). Each *sagon* sample is only repeated one time. The hedonic test analysis includes taste, color, aroma, and texture on a scale of 1–5 (11). Scale 1 represents the worst trait, while scale 5 represents the best trait.

### Shelf Life Estimation

This research was conducted with two experiments and two replications for water content, free fatty acid, and peroxide number. The shelf life was estimated using the Accelerated Shelf-Life Testing (ASLT) method with the Arrhenius equation, and the sample used was P2. P2 was chosen because, when compared to the other three treatments, it has the best organoleptic test findings. For this estimation, the sample was packed in a vacuum polypropylene plastic and stored in an incubator at 27, 37, as well as 47°C for 30 days, and the water content, peroxide number, as well as free fatty acid content were tested every 5 days. The product is kept at an accelerated temperature, with a minimum of three temperatures (27, 37, and 47°C) that can hasten product quality decline.

The moisture content was determined by using the oven method (9). The free fatty acid content was determined using the Lowrey and Tinsley method (12), and the peroxide value was determined using the Hornero-Méndez method and a xylenol orange color indicator. The parameter with the lowest activation energy value is used to determine the shelf life of *sagon* flour.

The slope (*k*), intercept (constant), and correlation coefficient (*R*^2^) of a graph of reaction kinetics for order zero or order one was plotted from the storage research to forecast deteriorating behavior. Do this for all the key elements you have chosen, such as quality parameters. For each storage temperature, the graph depicts the link between storage time (*x*-axis) and the value of quality parameters (*y*-axis). Next, compare all the specified factors to obtain the value of *k* for each storage temperature. As the temperature rises, the value of *k* (slope) will rise as well. Then, using an Arrhenius equation, show the relationship between 1/T in Kelvin (*x*-axis) and Ln *k* (*y*-axis) (for three observed temperatures). The value of *k* is calculated at the specified storage temperature or distribution. The rate of degradation per day at that temperature is the value of *k* in this equation. The following formula is used to compute the value of *k*:


$$\text{k}={\text{k}}_{0}.{\text{exp}}^{\left(-\text{Ea}/\text{RT}\right)}$$

Furthermore, the Arrhenius equation is used to calculate the estimated shelf life. Then, using the Arrhenius equation in zero and first order, the estimated shelf life of *sagon* is calculated as follows:


t=A⁢t-A⁢oK⁢(Ordo⁢ 0)⁢dan⁢t=L⁢n⁢(A⁢t)-L⁢n⁢(A⁢o)K⁢(Ordo⁢ 1)

where the *k* (degradation rate) is a value indicates the decrease of product quality; t, expired time; Ao, the initial concentration of the chemical of interest; At, the initial concentration of the chemical of interest at particular time “t.”

### Statistic Analysis

The effect of soybean flour on the nutrient content of *sagon* was determined through statistical analysis using one-way ANOVA followed by the Tukey and the Kruskal–Wallis tests and then by the Mann–Whitney test. In addition, the best formulation was determined using the Effectiveness Index (De Garmo) based on the nutritional content and organoleptic quality (13).

## Results and Discussion

### *Sagon* Nutrient Content of *Lindur* and Soybean Flour

#### Protein Content

The *sagon* protein levels were discovered to range from 8.78 to 11.45% and decreased with a reduction in the soybean flour content. In comparison with the emergency food standard provided by Zoumas et al. (3), the protein calorific contribution of formulations P1 to P4 is not in accordance with the emergency food standard of 10–15% protein 1. This is because *lindur* flour is low in protein 5.59% (14), while soybean is a source of vegetable protein with lower quality, compared to animal protein (15).

The low protein content is probably also due to heating during processing (Mallard reaction). The high content of amylose and amylopectin in starch has the capacity to facilitate the gelatinization process, leading to the hydrolyzation of *lindur* starch into reducing sugars. Subsequently, these reducing sugars bind to amino acids in soybean flour and produce volatile compounds, consequently, reducing the protein content (16, 17).

Furthermore, the use of temperatures above 115°C tends to facilitate Maillard reaction while the use of 230°C for 30 min has the capacity to cause a loss of 15% of amino acids (18). In this study, the *sagon* samples were oven-dried at 150°C for 20 min, to minimize protein and amino acids loss.

#### Fat Content

The *sagon* fat contents were discovered to range from 25.31 to 35.28%. In comparison with the emergency food standard provided by Zoumas et al. (3), the fat calorific contribution of formulation P1 comprising 45% *lindur* flour and 35% soybean flour, fulfilled the total fat standard of 35–45% (3). Meanwhile formulations P2, P3, and P4, exceeded the emergency food standard for fat content.

In a study by Thomas the addition of 25% soybean flour in the manufacture of banana flour biscuits led to a 15.29% increase in fat content (19). *Sagon* has a high-fat content of 28–30% due to the high-fat content of the coconut constituent. The high-fat content of *sagon* has the capacity to influence the product’s shelf life and increase the risk of rancidity due to lipid oxidation of the oil from coconut flesh. Fat oxidation is a major factor of degradation in food, therefore the free fatty acid parameter is used to determine the shelf life of foods with high fat content (20). This oxidative damage to lipids tends to cause undesirable tastes and odors during storage at room temperature of 27°C (21).

#### Carbohydrate Content

The *sagon* carbohydrate content was discovered to range from 48.81 to 60.18% and decreased with an increase in the soybean flour content. This is in line with the study by Dengo et al. (22), where the carbohydrate content of fish nuggets with mangrove flour substitution was reported to decrease with an increase in protein content and other substituted components (22). In comparison with the emergency food standard provided by Zoumas (3) the carbohydrate calorific contribution of formulations P1 to P3 fulfills the minimum calorie requirement of 40–50% (3). This calorific contribution is mainly due to *lindur* flour’s high carbohydrate content of 97.66% (23).

The addition of soy flour reduced the carbohydrate content. The protein, fat, and ash content of *sagon* increase as the proportion of soybean flour increases, while the carbohydrate content decreases. Conversely, as the amount of soy flour is reduced, the proportion of *lindur* flour increases, resulting in an increase in carbohydrates. Today’s emergency foods are high in energy and carbohydrates, with the goal of meeting sufficient energy needs while also providing a long-lasting satiety effect.

#### Water Content

The *sagon* water content was discovered to range from 4.04 to 2.46%, and formulation P1 to P4 all fulfilled the emergency food standard of below 14% (24). *Sagon* with a low water content can have a positive impact in emergency situations because it can inhibit the growth rate of microorganisms, resulting in a longer decay process and a longer shelf life. *Sagon*’s low water content is suspected because the raw materials used are already dry and have been dried, roasted, and baked. This study used a 150°C oven for 20 min. A temperature of 150°C, according to Jagat et al. (25), can reduce the water content in the material, resulting in a decrease in the water content of the product (25). According to Ayustaningwarno et al. (10), increasing temperature causes a decrease in water content (10).

#### Ash Content

The *sagon* ash content was discovered to range from 1.70 to 2.01%. According to Anandito et al. (26), the ash content of emergency food produced from flour ranges from 2 to 3% (26). Therefore, only formulation P4 comprising 30% *lindur* flour and 50% soybean flour, was in accordance with the emergency food quality requirements, while the ash contents of formulations P1 to P3 were below the emergency food standard.

*Sagon* ash content can be affected by the addition of soybean flour. The higher the addition of soybean flour to *sagon*, the higher the ash content. Soybeans are a high source of minerals. According to Jariyah and Pertiwi (27), the mineral sources contained in soybean flour are calcium, iron, copper, magnesium, and sodium (27). On the other hand, the addition of *lindur* flour can also contribute to increasing the ash content. *Lindur* flour contains several minerals such as calcium, phosphorus, zinc, potassium, magnesium, iron, sodium, and copper (5).

#### Food Fiber

The *sagon* soluble and insoluble dietary fiber contents were discovered to range from 1.01 to 1.48 and 14.78 to 17.64%, respectively. This is in line with the study by Bunyapraphatsara et al. (28), where the soluble and insoluble fiber content of *lindur* fruit flour was reported to be 3.13 and 14.8%, respectively. The high content of soluble and insoluble dietary fiber in *sagon* causes a high fiber content of 15.79–19.12%. However, adequacy of fiber is obtained by adjusting the total dietary fiber content to 10% of the daily requirement (30 g/day), which is 3 g/day (29).

According to the regulation of the head of the Food and Drug Supervisory Agency, 13 Number 2016, concerning the supervision of claims on processed food labels and advertisements, a product is classified as high fiber if the fiber content is greater than 6 g per 100 g of sample (30). As a result, the *sagon* produced in this study has a high dietary fiber content, which has a positive impact on victims of natural disasters, such as long-lasting satiety, digestion facilitation, prevention of constipation and hemorrhoids, and health improvement (31).

#### Energy Content

The *sagon* calorific value was discovered to range from 251.53 to 278 kcal. In comparison with the emergency food standard, formulations P1 and P2 fulfill the emergency food requirements of 233–250 kcal/bar, while P3 and P4 exceed the requirements by 7.8 and 11.2%, respectively. Therefore, the *sagon* ingredients are an adequate source of energy for emergency conditions. The energy levels in *sagon lindur* flour and soybean flour increased. The more *lindur* and soybean flour that is added, the higher the energy content of *sagon*. It can be concluded that the ingredients used to make *sagon* contribute as a source of emergency food standards. Table 2 shows the results of the proximate analysis of formulations P1 to P4. Table 3 shows the calorific contribution of *sagon* macronutrients from *lindur* and soybean flour.

**TABLE 2:** The results of proximate analysis of *sagon* formulated with *lindur* and soybean flour.

Nutrient content/100 g (mean ± SD)				

Nutrients	Treatment			
	P1	P2	P3	P4
Energy (kcal)*	503.06 ± 19.46^a^	514.44 ± 5.5^b^	540.52 ± 18.37^c^	557.07 ± 14.47^c^
Proteins (grams)**	8.78 ± 0.06^a^	9.30 ± 0.03^b^	9.82 ± 0.03^c^	11.45 ± 0.20^d^
Fat (grams)*	25.31 ± 3.81^a^	27.53 ± 0.98^b^	31.94 ± 3.80^c^	35.05 ± 2.76^c^
Carbohydrates (grams)*	60.06 ± 3.91^a^	57.35 ± 0.89^ab^	53,45 ± 3.97^bc^	48.95 ± 2.56^c^
Water content (% wb)**	4.03 ± 0.16^a^	3.98 ± 0.14^b^	2.86 ± 0.39^c^	2.46 ± 0.08^d^
Ash content (% wb)*	1.84 ± 0.10^a^	1.84 ± 0.11^a^	1.93 ± 0.40^ab^	2.09 ± 0.12^c^
Crude fiber (%)*	7.62 ± 0.41	10.12 ± 3.32	12.64 ± 1.05	12.25 ± 2.99
Soluble dietary fiber (% wb)*	1.01 ± 0.08^a^	1.24 ± 0.03^b^	1.36 ± 0.00^c^	1.48 ± 0.06^d^
Insoluble dietary fiber (% wb)*	14.78 ± 0.18^a^	15.39 ± 0.10^b^	16.74 ± 0.06^c^	17.64 ± 0.13^d^
Total dietary fiber (% wb)*	15.79 ± 0.21^a^	16.63 ± 0.11^b^	18.10 ± 0.07^c^	19.12 ± 0.07^d^

Numbers followed by different superscript letters (a, b, c, and d) indicate a significant difference. *Testing with one-way ANOVA. **Testing with Kruskal–Wallis test. n = 12 (4 experiments × 3 replications) for proximate analysis. P1 (lindur 45%: soybean flour 35%), P2 (lindur 40%: soybean flour 40%), P3 (lindur 35%: soybean flour 45%), and P4 (lindur 30%: soybean flour 50%).

**TABLE 3:** Caloric contribution of *sagon* formulated with *lindur* and soybean flour.

Macronutrient caloric contribution					

Nutrients	P1	P2	P3	P4	Emergency food standard
Proteins (%/50 g)	6.98	7.242	7.29	8.23	10–15%^a^
Fat (%/50 g)	45.28	49.46	53.40	56.72	35–45%^a^
Carbohydrates (%/50 g)	47.74	45.78	39.66	35.20	40–50%^a^
Total calories (kcal/50 g)	251.53	257.22	270.34	278.54	233–250 kcal^a^
Ash content (%)	1.85	1.84	1.93	2.09	2–3%^b^
Water content (%)	4.03	3.98	2.86	2.46	<14%^a^
Dietary fiber (%)	15.79	16.63	1.10	19.12	>6 g/100 g^c^

*P1 (lindur 45%: soybean flour 35%), P2 (lindur 40%: soybean flour 40%), P3 (lindur 35%: soybean flour 45%), and P4 (lindur 30%: soybean flour 50%). ^a^Zoumas et al. (3), ^b^Anandito et al. (26), c = BPOM (30).*

### Organoleptic Evaluation

Generally, the most preferred *sagon* formulation was formulation P2 comprising 40% *lindur* flour and 40% soybean flour. Table 4 shows the results of the organoleptic evaluation of *sagon* formulated with *lindur* and Soybean flour. Based on the color analysis, the most preferred formulation was P4 comprising 50% soybean flour substitution, and with a light brown color. In this study, the *sagon* sample color tended to improve with reduction in *lindur* flour content, with formulation P1 having the least attractive color (dark brown to slightly blackish) (6).

**TABLE 4:** The results of the organoleptic evaluation of *sagon* formulated with *lindur* and soybean flour.

Parameter	Organoleptic quality (mean ± SD)			
	Treatment			
	P1	P2	P3	P4
Color	3.60 ± 0.77^b^ (brown)	4.13 ± 0.63^a^ (brown)	4.10 ± 0.66^a^ (brown)	4.37 ± 0.49^a^ (brown)
Taste	4.07 ± 0.83^a^ (not bitter)	4.17 ± 0.75^a^ (not bitter)	3.77 ± 0.94^a^ (not bitter)	3.90 ± 0.85^a^ (not bitter)
Aroma	3.87 ± 0.63^a^ (odorless)	3.80 ± 0.76^a^ (odorless)	3.57 ± 0.97^a^ (odorless)	3.57 ± 0.97^a^ (odorless)
Texture	2.87 ± 0.86^b^ (slightly fragile)	2.97 ± 0.99^ab^ (slightly fragile)	3.10 ± 1.29^ab^ (slightly fragile)	3.63 ± 0.96^a^ (slightly fragile)

*Numbers followed by different superscript letters (a and b) show a significant difference with the Kruskal–Wallis testing. P1 (lindur 45%: soybean flour 35%), P2 (lindur 40%: soybean flour 40%), P3 (lindur 35%: soybean flour 45%), and P4 (lindur 30%: soybean flour 50%). N = 12 (4 experiments × 3 replications) for organoleptic evaluation.*

The organoleptic quality of *sagon* color is influenced by the substitution of *lindur* and soybean flour. The score of the organoleptic quality test of *sagon* color tends to increase as the concentration of *lindur* flour added decreases. *lindur* flour has a natural brown color that is influenced by processing as well as the presence of pigments such as chlorophyll, carotenoids, anthocyanins, anthoxanthins, and tannins (5). Furthermore, the drying stage during flour-making has the capacity to cause tannin oxidation, and the tannin content tends to produce a yellow or brown coloration, consequently, darkening the flour’s color (32).

The formation of brown color is also caused by an enzymatic browning reaction. According to Harrison and Dake (33), the enzymatic browning reaction of phenolic compounds is mostly catalyzed by the oxygenase enzyme in the form of polyphenol oxidase released during the material’s exposure. This process is initiated by the hydroxylase reaction of monophenol to produce diphenol which undergoes oxidation to produce quinone, consequently, producing dark, yellow, orange, and brown coloration (33).

A study by William in Sulistyawati et al. (32), showed brown coloration also occurs due to non-enzymatic reactions due to heating. This process leads to the Maillard reaction, a browning reaction that occurs between carbohydrates (reducing sugars) and amino acids (32). The carbohydrate content of *lindur* flour, as well as the protein content of soybean flour, tend to facilitate this reaction, leading to brown *sagon* products and this is an indication of a reduction in product quality (34).

The panelists preferred the sweet taste of *sagon*. *Sagon*’s popularity is decreasing because it has a bitter aftertaste, which is thought to be caused by the tannin content of *lindur* flour (35). Tannins are acidic polyphenolic compounds with an astringent taste, and these compounds tend to cause a bitter taste in *lindur* fruit flour (36). However, in this study, efforts were made to reduce the bitter taste by soaking (37).

Similarly, soybean flour has a bitter taste caused by the presence of glycoside compounds, particularly saponin, which has relatively heat-resistant properties and is, therefore, difficult to remove (38, 39). In addition, soybeans contain lipoxygenase enzymes, and these hydrolyze or decompose polyunsaturated fatty acids, consequently, producing compounds with unpleasant odors, especially ethyl phenyl ketone (38).

In this study, formulations P3 and P4 were discovered to have a slightly unpleasant aroma which increased with an increase in the soy flour content, consequently influencing the resulting product quality. Because of the presence of the lipoxygenase enzyme, soybean flour has a distinct aroma. Lipoxygenase enzymes can hydrolyze or decompose polyunsaturated fatty acids, resulting in the formation of compounds that emit unpleasant odors, particularly ethyl phenyl ketone (38). However, the lipoxygenase enzyme is rendered ineffective by soaking and heating during the manufacture of the *sagon* (40). The addition of sweeteners and flavoring also help eliminate any unpleasant aromas and tastes (41).

Formulations P1, P2, and P3 have a slightly brittle texture due to the high amylopectin contents of *lindur* flour. According to Bunga et al. (16), the amylopectin content of *lindur* flour is 80.506% (16). An increase in the amylopectin content implies lower starch solubility and water absorption (42). Starch granules in suspension absorb water, swell, and eventually solubilize during gelatinization. Fully gelatinized starch granules will result in better starch cell rupture during the gelatinization process. The amylose to amylopectin ratio of the flour influences the degree of expansion of the product. The expansion was reduced because less water was trapped in the network of starch gel. According to Maisont et al. (43), the lower the degree of cracker expansion, the fewer air cells formed and trapped, and thus the lower the cracker hardness (43).

Formulation P4 was discovered to possess the least crumbly texture, influenced by high fiber and protein content. Fiber has a bulking ability which produces a denser texture, while protein has hydrophilic properties with the ability to absorb high amounts of water, consequently, increasing the product’s density and sturdiness (44, 45).

### Determination of the Best Formulation

This was carried out using the product effectiveness test based on the De Garmo method (13). The calculation begins with determining the weight of the variable with a scale of 0–1 on each parameter based on the highest priority starting from the content of energy (1), protein (0.9), fat (0.8), carbohydrates (0.7), dietary fiber (0.6), crude fiber (0.5), organoleptic quality (0.4), preference level (0.3), moisture content (0.2), and ash content (0.1). Then determine the value of effectiveness (Ne) on each variable. The last step is to calculate the result value (Nh) for each variable obtained. The best formulation chosen is the formulation that has the highest total Nh from adding up all Nh variables.

According to the results, the highest total yield value (Nh) of all variables was found in formulation P1 comprising 45% *lindur* flour and 35% soybean flour. Table 5 shows the results of the determination of the best formulation. The best formulation chosen is the formulation that has the highest total Nh. The best formulation is determined by considering all aspects of the parameters, specifically the content of nutrients (carbohydrates, fat, protein, dietary fiber, crude fiber, water content, and ash content), dietary fiber, and hedonic quality sagon that are close to the emergency food.

**TABLE 5:** The results of the determination of the best formulation.

Formula/yield value (Nh)*	P1	P2	P3	P4
Energy	0.1818	0.0871	0.0461	0.0000
Protein	0.0000	0.0318	0.0637	0.1636
Fat	0.1455	0.1122	0.0461	0.0000
Carbohydrate	0.1273	0.0965	0.0513	0.0000
Total dietary fiber	0.0000	0.0276	0.0756	0.1091
Coarse fiber	0.0909	0.0457	0.0000	0.0070
Water content	0.0000	0.0024	0.0541	0.0727
Ash content	0.0019	0.0000	0.0204	0.0545
Organoleptic quality	0.0000	0.0227	0.0045	0.0364
Hedonic test	0.0074	0.0182	0.0050	0.0000
Total of yield value (Nh)	0.5547	0.4443	0.3669	0.4434

*The yield value (Nh) is obtained from the De Garmo effectiveness index. n = P1 (lindur 45%: soybean flour 35%), P2 (lindur 40%: soybean flour 40%), P3 (lindur 35%: soybean flour 45%), and P4 (lindur 30%: soybean flour 50%).

This formulation’s energy content, carbohydrates, fat, water content, ash content, and dietary fiber are in accordance with the emergency food quality requirements, however, the protein content is deficient. The more lindur fruit flour added, the higher the carbohydrate, energy, and water content. The addition of lindur flour is used to highlight and utilize the basic constituent of lindur (*B. gymnorrhiza* L.), which is widely available in underdeveloped coastal areas. *Lindur* flour contains high energy, carbohydrates, and fiber, but the protein content of *lindur* is low, therefore, to increase the protein value, it can be increased by adding soybeans. The addition of soy flour as much as 35% have been through consideration correct, if you add too much soy flour, the fat content will increase so that the fat content exceeds the emergency food quality requirements. According to Efraim’s (?) research, adding 25% soybean flour to banana flour biscuits can boost fat content by 15.29% (19). Furthermore, the higher the proportion of soy flour added, the increased the protein, fat, and ash content of sagon, but the carbohydrate content decreased, and the emergency food quality standards were not met.

### Moisture Kinetic

**FIGURE 1:**
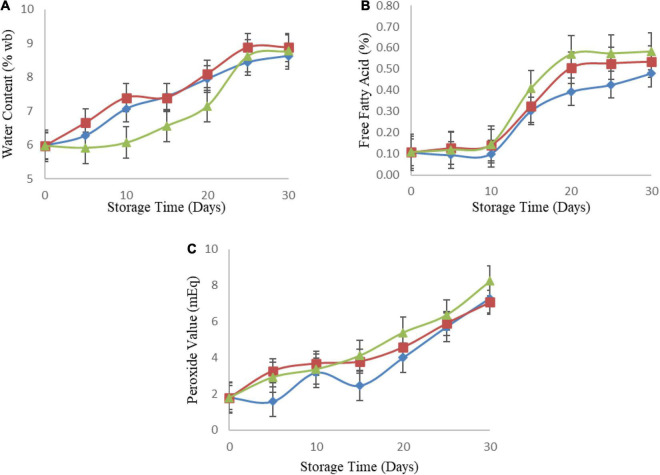
Change in water content (%wb) **(A)**, free fatty acid (%) **(B)**, peroxide value (mEq) **(C)**, during storage at 27, 37, and 47°C. (◆ = 27°C), (■ = 37°C), and (▲ = 47 °C). Error bar is standard error. *n* = 4 (2 experiments × 2 replications) for water content, free fatty acid and peroxide number.

The water content test was the parameter tested in the study of estimating the shelf life of *sagon*. Observations were made with three treatments, namely temperatures of 27, 37, and 47°C for 30 days with an observation time span of every 5 days, resulting in a graph with seven observation points, as shown in Figure 1.

The values of 1/T and ln *k* were plotted and the linear regression equation y = −596.25x – 0.3965, as well as *R*^2^ of 0.7779, were obtained. Based on linear regression analysis of the graph above, the equation of the line y = −596.25x – 0.3965 with *R*^2^ = 0.7779, was obtained. Subsequently, the slope of this equation was used to determine the activation energy for the water content which was 1.18 kcal/mol.

*Sagon* is processed by baking and is, therefore, hygroscopic or able to absorb water easily, meaning the water content tends to increase during storage (46). According to Gichau et al.’s (47), there are three significant interactions (*p* < 0.005) that indicate that the type of packaging, storage time, and storage conditions can affect the water content of Amaranth Sorghum grains based Complementary Food (ASCF) products. High water content has the capacity to cause enzymatic hydrolyzation of fat (47), consequently, accelerating the deterioration of product quality and shortening the shelf life.

### Free Fatty Acid Kinetic

The test of free fatty acid levels was another parameter analyzed in the study on estimating the shelf life of *sagon*. Carried out with three treatments, such as temperatures of 27, 37, and 47°C for 30 days with a 5-day observation interval to obtain seven observation points. Figure 1 shows a graph of the results of observations of changes in free fatty acid.

The values of 1/T and ln *k* were plotted and the linear regression equation y = −1392.4x – 4.1696, as well as an R^2^ of 0.996, were obtained. Based on the graph above, the equation of the line y = −1392.4x – 4.1696 with R^2^ = 0.996, was obtained. Subsequently, the slope value of the equation was used to determine the activation energy for the free fatty acid content which was 2.77 kcal/mol.

**FIGURE 2:**
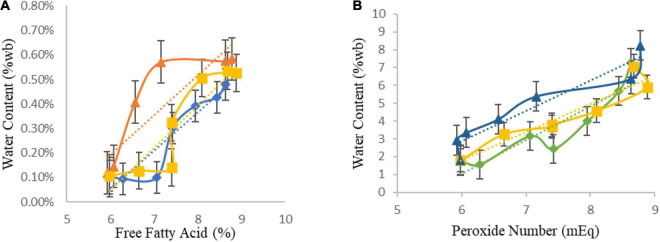
The relationship between water content and free fatty acid **(A)**, the relationship between water content and peroxide number **(B)**, during storage at 27, 37, and 47°C. (◆ = 27°C), (■ = 37°C), and (▲ = 47°C). Error bar is standard error. *n* = 4 (2 experiments × 2 replications).

The free fatty acid levels were observed to increase daily until the end of storage on the 30th day, with the highest value of 0.58% discovered in the sample stored at 47°C. The changes in free fatty acid levels indicate the occurrence of fat hydrolysis occurs in the *sagon*. However, this value is below the Indonesian National Standard for Biscuit Quality SNI No. 01-2973-2011 of 1.0% maximum free fatty acid. Therefore, the free fatty acid levels after storage for 30 days at all temperatures are suitable for consumption. Figure 2 shows a graph of the relationship between water content and free fatty acid.

The increase in free fatty acid levels during storage was caused by hydrolysis of fat, and this process is influenced by the water content which will convert fat into free fatty acids and glycerol. Therefore, an increase in the product’s water content is bound to increase the rate of lipid auto-oxidation, consequently, reducing the product quality (48). Based on the Figure 2, the relationship between water content and free fatty acid levels had a correlation *R*^2^ of 0.996, implying the relationship between water content and free fatty acid content is linear. This is in accordance with the study by Pertiwi et al. (48), where a linear relationship was reported between the water content and free fatty acid content.

The highest free fatty acid levels were found in *sagon* stored at 47°C, which was 0.58%. High temperature causes the decomposition of unsaturated fatty acids to decompose, consequently, breaking the double bond chain. This increases the free fatty acid levels, while the broken chain will bind to oxygen, thus, accelerating lipid oxidation and causing unwanted aroma (49).

### Peroxide Value Kinetic Parameters

The peroxide number was another parameter analyzed in the study on estimating the shelf life of *sagon*. Carried out with three treatments, such as temperatures of 27, 37, and 47°C for 30 days with a 5-day observation interval to obtain seven observation points. Figure 1 shows a graph of the results of observations of changes in peroxide number.

The values of 1/T and ln *k* were plotted and the linear regression equation of y = −472.27x – 0.1934, as well as an *R*^2^ of 0.1507, were obtained. Based on the graph above, the equation of the line y = −472.27x – 0.1934 with *R*^2^ = 0.1507 was obtained. Subsequently, the slope value of the equation was used to determine the activation energy of the water content which was 0.94 kcal/mole.

Fat damage is distinguished by the presence of a rancid aroma and taste, also known as rancidity. Rancidity occurs as a result of enzymatic and non-enzymatic oxidation and hydrolysis reactions. By determining the peroxide value, this parameter can be used to determine the decrease in *sagon* quality caused by fat breakdown (46).

The *sagon* peroxide number was discovered to increase daily until the end of storage on the 30th day. This is caused by accelerated oxidation which is influenced by humidity, temperature, and oxygen. Based on the results by Nurhasnawati et al. (50), each 10°C rise in temperature doubles the rate of oxidation. This is in line with the study by 2, where the peroxide number was reported to increase 0.49 mEq/Kg during storage at three temperatures.

According to the results, the *R*^2^ of 0.1507 has a value of about 1, indicating a linear relationship between water content and peroxide number. Figure 2 shows a graph of the relationship between water content and peroxide number. This is in line with the study by Akinoso et al. (51), where a significant relationship was reported between the water content, roasting time, and peroxide value. This is because the water content has the capacity to accelerate the fat oxidation reaction and consequently, increasing the peroxide value. The products of oxidation result in rancid taste, as well as changes in odor, leading to a reduction in nutritional value, due to vitamins and essential fatty acids damage (52).

### Shelf Life

This research was conducted with two experiments and two replications, and the sample used was P2. P2 was chosen because, when compared to the other three treatments, it has the best organoleptic test findings. *Sagon* packed in PP plastic with a vacuum and a thickness of 80 μm was stored in an incubator for 30 days at temperatures of 27, 37, and 47°C with an observation period of once every 5 days to obtain 7 observation points. The parameters of water content, free fatty acid content, and peroxide number were used in this study.

Based on the Arrhenius plot, an equation was obtained, and this was used to determine the activation energy (Ea). Table 6 shows comparison of the order of water content parameters, free fatty acid, peroxide number, and activation energy. The estimation of shelf life was carried out by determining the initial value, critical value, and decreasing rate of quality. Table 7 shows the shelf life at each temperature.

**TABLE 6:** Comparison of the order of water content parameters, free fatty acid, peroxide number, and activation energy.

Test parameter	Temperature (°C)	Regression equation		*R* ^2^		Activation energy Ea (kcal/mol)
				
		Ordo 0	Ordo 1	Ordo 0	Ordo 1	
Water content	27	y = 0.0939x + 5.9875	y = 0.0129x + 1.7988	0.9843	0.9751	1.18
	37	y = 0.0946x + 6.166	y = 0.0127x + 1.8265	0.9423	0.9349	
	47	y = 0.1065x + 5.4194	y = 0.0148x + 1.7139	0.8711	0.8893	
Free fatty acid	27	y = 0.0001x + 0.0005	y = 0.0635x − 7.0748	0.9007	0.8456	2.77
	37	y = 0.0002x + 0.0006	y = 0.064x − 6.8981	0.8997	0.8989	
	47	y = 0.0002x + 0.0006	y = 0.0689x − 6.9056	0.8785	0.8679	
Peroxide number	27	y = 0.1822x + 0.9674	y = 0.0501x + 0.417	0.8650	0.8900	0.94
	37	y = 0.157x + 1.9421	y = 0.0394x + 0.7886	0.9434	0.9140	
	47	y = 0.02x + 1.5675	y = 0.0472x + 0.7096	0.9778	0.9680	

**TABLE 7:** Estimation of *sagon* shelf life* (54).

Temperature (°C)	Initial value of quality (mEq/Kg)	Critical value of quality (mEq/Kg)*	Decreasing rate of quality (K)	Shelf life (days)
27	1.792	8.00	0.1707	37
37	1.792	8.00	0.1796	35
47	1.792	8.00	0.1884	33

The product is stored at room temperature of 27°C, therefore, the estimated shelf life is 37 days. According to the results, all parameters have a zero-order reaction, because the value of *R*^2^ is zero-order > first order, therefore, the shelf life was estimated using the regression of zero order. Furthermore, the parameter with the least activation energy value was used because the activation energy A reduction in activation energy implies a faster reaction time, and consequently, a faster reduction in product quality (53). In this study, the parameter with the least activation energy was discovered to be the peroxide number. The critical value of quality was obtained from the criteria of biscuits according to SNI No. 01-2973-2011 (54). For the peroxide number parameter of 8 mEq/kg. This is not in accordance with the standard criteria for emergency food shelf life, which ought to be a minimum of 36 months at 21°C (55).

In this study, samples were stored at 27, 37, and 47°C. Every 10°C increase in temperature can hasten the deterioration of food quality during storage (20). Because chemical reactions occur at a faster rate at higher temperatures, product quality degrades faster. As a result, at a temperature of 47°C, the shelf life was shorter than at a temperature of 27°C. This is consistent with the findings of the (53) study, which found that the higher the storage temperature, the shorter the shelf life of apple pie. According to this study, an increase in temperature causes an increase in the reaction rate, which causes apple pie to spoil quickly, resulting in a shorter shelf life. As a result, it is recommended for *sagon* storage.

## Conclusion

The energy, carbohydrate, fat, water, ash, and dietary fiber content of P1 are all within the emergency food quality requirements, but the protein content is still below the emergency food quality requirements. The more *lindur* fruit flour added, the higher the carbohydrate, energy, and water content. Protein, fat, and ash content, on the other hand, will decrease. The lower the protein and ash content, the less soy flour is used. Formulation P1 comprising 45% *lindur* flour and 35% soybean flour was concluded to be the best formulation. This formulation’s energy content, carbohydrates, fat, water content, ash content, and dietary fiber are in accordance with the emergency food quality requirements, however, the protein content is deficient. In addition, the shelf life of P2 *sagon* formulated with *lindur* and soybean flour and stored in polypropylene packaging was estimated to be 37 days at room temperature of 27°C, using the ASLT method.

## Data Availability Statement

The original contributions presented in the study are included in the article/supplementary material, further inquiries can be directed to the corresponding author.

## Ethics Statement

The studies involving human participants were reviewed and approved by the Health Research Ethics Committee, Faculty of Medicine, Diponegoro University with ethical clearance No. 119/EC/KEPK/FKUNDIP/VI/2021. The patients/participants provided their written informed consent to participate in this study.

## Author Contributions

DA, FA, NN, DS, YN, and TS wrote the manuscript. DA, FA, NN, and DS conceptualized and designed the study, collected the data, analyzed and interpreted the data, and performed the manuscript drafting and critical revision. YN and TS carried out the research, data collection and data analysis and interpretation. DA revised the manuscript critically. All authors contributed to the revision of the manuscript and read and approved the final version.

## Conflict of Interest

The authors declare that the research was conducted in the absence of any commercial or financial relationships that could be construed as a potential conflict of interest.

## Publisher’s Note

All claims expressed in this article are solely those of the authors and do not necessarily represent those of their affiliated organizations, or those of the publisher, the editors and the reviewers. Any product that may be evaluated in this article, or claim that may be made by its manufacturer, is not guaranteed or endorsed by the publisher.
